# Diethyl aminoethyl hexanoate reprogramed accumulations of organic metabolites associated with water balance and metabolic homeostasis in white clover under drought stress

**DOI:** 10.3389/fpls.2024.1430752

**Published:** 2024-10-11

**Authors:** Muhammad Jawad Hassan, Atiqa Najeeb, Min Zhou, Muhammad Ali Raza, Ummar Ali, Bizhen Cheng, Yao Ling, Zhou Li

**Affiliations:** ^1^ College of Grassland Science and Technology, Sichuan Agricultural University, Chengdu, China; ^2^ Institute of Soil Fertilizer and Water Saving Agriculture, Gansu Academy of Agricultural Sciences, Lanzhou, China; ^3^ State Key Laboratory of Crop Gene Exploration and Utilization in Southwest China, Sichuan Agricultural University, Chengdu, China

**Keywords:** water deficit, metabolomics, organic acids, sugars, physiological function, photosynthetic performance

## Abstract

Diethyl aminoethyl hexanoate (DA-6) serving as a non-toxic and low-cost plant growth regulator is used for improving plant growth and stress tolerance, but the DA-6-mediated organic metabolites remodeling in relation to drought tolerance is not well documented in crops. The aims of the present study were to evaluate impacts of DA-6 on physiological functions including osmotic adjustment, photochemical efficiency, oxidative damage, and cell membrane stability as well as organic metabolites remodeling in white clover (*Trifolium repens*) leaves based on the analysis of metabolomics. Plants were foliarly treated with or without DA-6 and subsequently exposed to drought stress for 8 days. Results demonstrated that foliar application of DA-6 (1.5 mM) could significantly ameliorate drought tolerance, which was linked with better leaf water status, photosynthetic performance, and cell membrane stability as well as lower oxidative injury in leaves. Metabolic profiling of organic metabolites identified a total of 59 metabolites including 17 organic acids, 20 sugars, 12 alcohols, and 10 other metabolites. In response to drought stress, the DA-6 induced accumulations of many sugars and sugar alcohols (erythrulose, arabinose, xylose, inosose, galactose, talopyranose, fucose, erythritol, and ribitol), organic acids (propanoic acid, 2,3-dihydroxybutanoic acid, palmitic acid, linolenic acid, and galacturonic acid), and other metabolites (2-oxazoline, silane, and glycine) in white clover. These altered metabolites induced by the DA-6 could perform critical functions in maintenances of osmo-protection, osmotic adjustment, redox homeostasis, cell wall structure and membrane stability when white clover suffered from water deficit. In addition, the campesterol and stigmasterol significantly accumulated in all plants in spite of the DA-6 pretreatment under drought stress, which could be an important adaptive response to water deficit due to beneficial roles of those two metabolites in regulating cell membrane stability and antioxidant defense. Present findings provide new evidence of DA-6-regulated metabolic homeostasis contributing to drought tolerance in leguminous plants.

## 1 Introduction

Due to global warming, the world is experiencing serious environmental problems including increased CO_2_ level and drought (Gosling and Arnell, 2016; Jackson et al., 2017). Among these, drought is deliberated as the chief environmental issue with severe agricultural penalties (Fahad et al., 2017), because water scarcity significantly disrupts plant water status and also induces stomatal closure resulting in decreased CO_2_ assimilation, carbon fixation, and photosynthesis for ATP production (França et al., 2000; Flexas and Medrano, 2002; Wu et al., 2008; Liang et al., 2019). In addition, drought stress disturbs the redox homeostasis leading to excessive reactive oxygen species (ROS), which induces severe oxidative injury to functional biomolecules such as nucleic acids, chlorophylls, proteins, and membrane lipids, thereby promoting programmed cell death (Gill and Tuteja, 2010; Sharma et al., 2019). However, undesirable consequences induced by drought stress depend on various factors such as the duration or intensity of the stress, plant growth stage, and species or genotype (Okçu et al., 2005; Hassan et al., 2022). Plants tend to acclimatize hazardous conditions by regulating morphological, biochemical, and physiological responses at genetic and metabolic levels (Gong et al., 2020; Li et al., 2021; Zhang et al., 2021).

By metabolomics analysis, biologically important and significantly different metabolites are identified and separated from specific cells or tissues, and different metabolic processes as well as response mechanisms occurring in organisms are studied (Sumner et al., 2003). Plant metabolomics has become one of the main research hot spots in plant-based studies and serves as an indispensable bridge linking plant phenotypes and genotypes (Hall, 2006). Many studies have shown that the accumulation and reprogramming of comprehensive metabolites are not only related to quality and quantity of crops, but also contributed to alteration of stress tolerance (Khan et al., 2019; Patel et al., 2022; Zhou et al., 2023). Some of them are advantageous for increased stress tolerance such as soluble sugars and multiple amino acids including proline, glycine, and γ-aminobutyric acid etc., while other metabolites such as aldehydes and quinones in massive quantity are harmful for plants under unfavorable environmental conditions (Srivastava et al., 2017; Martínez-Noël and Tognetti, 2018; Batista-Silva et al., 2019). Global metabolites reprogramming has been reported to be associated with drought tolerance in different plant species based on comparative metabolomics (Sanchez et al., 2012). Moreover, spermine-regulated accumulation of organic metabolites such as galactose, sucrose-6-phosphate, mannose, and maltose enhanced heat or drought tolerance of creeping bentgrass (*Agrostis stolonifera*) by using non-targeted metabolomics (Li et al., 2022). Thus, study on relationship between alterations in metabolites profile and stress tolerance is crucial for broad-spectrum insight about stress responsive mechanisms in different plant species.

Over the last decade, the use of plant growth regulators (PGRs) for ameliorating crop yield and stress tolerance in the agricultural sector has become a common practice worldwide (Khan et al., 2020). Diethyl aminoethyl hexanoate (DA-6) is an important synthetic tertiary amine with multiple beneficial effects in plants under normal and stressed conditions (Hassan et al., 2021a). For example, DA-6 has been reported to enhance germination and seedling establishment of aged soybean (*Glycine max*) seeds via regulation of fatty acid metabolism and glycometabolism (Zhou et al., 2019). Foliar application of DA-6 after anthesis significantly mediated wheat (*Triticum aestivum*) grain filling, hence contributing to improved crop production (Wen et al., 2019). DA-6 also improved grain yield of summer maize (*Zea mays*) by increasing leaf photosynthetic functions and defense-related enzymes activities under field conditions (Nie et al., 2010). Moreover, the DA-6-regulated mechanisms of the tolerance to abiotic stress have been shown in many plant species. For example, the study of (Hassan et al., 2022) found that DA-6 ameliorated white clover (*Trifolium repens*) seedlings growth by regulating oxidative injury, photosynthetic performance, and lipids reprogramming under water deficient condition. DA-6 significantly strengthened antioxidant defense system in plants under different abiotic stresses such as low temperature and heavy metal toxicity (Fu et al., 2011; He et al., 2013; Li et al., 2018). Exogenous application of DA-6 effectively alleviated salt-induced oxidative damage in *Cassia obtusifolia* (Zhang et al., 2016). Foliar supplementation of DA-6 performed a positive function in increasing cadmium-extraction efficiency, thereby alleviating cadmium stress (He et al., 2014). The study of (Hassan et al., 2021a) also reported that DA-6 enhanced germination of white clover seeds by enhancing osmotic adjustment (OA), hormonal homeostasis, and accumulation of dehydrins under drought stress. Regardless of these previous studies, the mediatory function of DA-6 in drought tolerance in relation to organic metabolites remodeling still requires to be further investigated in plants.

White clover occupies a core position among global cool-season forage crops because of highly palatable, easily digestible, and nutritionally rich traits as well as tremendous ability to fix nitrogen in soils, hence contributing significantly to soil fertility and livestock industry (Cranston et al., 2015; Caradus et al., 2023; Sawicka et al., 2023). It’s also used as an important ornamental grass and ground cover plant for urban landscape (Sincik and Acikgoz, 2007). However, ineffective transpirational regulation and shallow root system make white clover highly vulnerable to water stress (Annicchiarico and Piano, 2004). Thus, the improvement of drought tolerance of legume crops like white clover is crucial to increase the production and quality of forage and ruminant’s performance. Aims of the study were to elucidate the impact of foliar DA-6 spray on oxidative damage, water status, and photochemical efficiency and to further reveal metabolic balance associated with organic metabolites reprogramming based on metabolomics in white clover plants exposed to drought stress. Current findings will supply vital information about regulatory roles and mechanisms of the DA-6 in legume species in response to drought stress.

## 2 Materials and methods

### 2.1 Planting materials & treatments

White clover seeds cv. ‘Haifa’ were sterilized with mercuric chloride solution (0.1%) for 4 min and washed twice with deionized water. Sterile 0.05 g of seeds were uniformly sown in each plastic box (18 cm breadth, 24 cm length, or 9 cm deep) containing sterilized quartz sands in a controlled environment (700 μmol photon m^−2^ s^–1^ PAR, 23/19°C day/night temperature, 12 h photoperiod cycle, and a relative humidity of 65%). Seeds were irrigated initially with distilled water for 7 days of germination, and then the distilled water was replaced by half-strength Hoagland solution (Hoagland and Arnon, 1950) until the second leaf expanded completely. Before being exposed to drought stress, one-month-old seedlings were pretreated by foliar application of DA-6 (1.5 mM) or deionized water once a day for 3 consecutive days to ensure that plants could absorb adequate quantity of DA-6 or deionized water through leaves. DA-6-treated and untreated plants were then exposed to Hoagland’s solution (control) or drought stress (-0.3 Mpa) induced by 17% polyethylene glycol (PEG) 6000 which was dissolved in Hoagland’s solution for the next 8 days. All solutions were refreshed daily to prevent any change in concentration. Four different treatments were used for this experiment: 1) C, plants grown in Hoagland’s solution as well-watered control; 2) C+DA-6, DA-6-pretreated plants grown in Hoagland’s solution; 3) PEG, plants were cultivated in -0.3 Mpa PEG solution as drought stress; 4) PEG+DA-6, DA-6-pretreated plants were cultivated in -0.3 Mpa PEG solution. All treatments comprised of four biological replicates and were arranged in a completely randomized design (CRD) in growth chamber. Leaf samples were taken on the 8^th^ day of drought stress for analyses of physiological parameters and metabolomics. The optimum dose of DA-6 (1.5mM) was chosen based on the result of our previous study (Hassan et al., 2022).

### 2.2 Measurements of leaf water status & osmotic adjustment

To determine leaf relative water content (RWC), fresh leaves (0.1g) were cut and promptly weighed to note the fresh weight (FW). Afterwards, leaves were submerged in deionized water for 1 day to attain the turgid weight (TW). Samples were then dried by placing them in an oven at 80°C for three consecutive days, and dry weight (DW) was measured. The formula (RWC (%) = 100×[(FW-DW)/(TW-DW)]) devised by (Barrs and Weatherley, 1962) was utilized to calculate leaf RWC. To evaluate osmotic potential (OP), leaf tissues were detached and dipped in deionized water for 8 h to ensure sufficient hydration. The hydrated leaves were taken out, blotted dry, and kept in liquid nitrogen for further analysis. After being thawed in an ice bath, leaf sap was extricated. A 10 μl leaf sap was taken and injected into an osmometer (Wescor, Inc., Logan, UT) to estimate osmolality (mmol kg^−1^). OP was calculated by using the following formula: MPa = −osmolality × 0.001 × 2.58 (Blum, 1989).

### 2.3 Measurements of chlorophyll content and photochemical efficiency

For chlorophyll (Chl) content, fresh leaf samples (0.1 g) were dipped in 15 ml of dimethyl sulphoxide and placed in dark environment for two days. Afterwards, leaf extract was collected, and the absorbance value was noticed spectrophometrically at 663 or 645 nm. The formula defined by (Arnon, 1949) was used for computing contents of Chl a, chl b, and total Chl. For determination of photochemical efficiency (Fv/Fm) and performance index on an absorption basis (PIBAS), fresh leaves were subjected to dark conditions with leaf clips for half an hour. Later, the Fv/Fm ratio and PIABS was noticed by using a fluorescence meter (Pocket PEA, Hansatech, United Kingdom).

### 2.4 Measurements of electrolyte leakage & oxidative damage

Electrolyte leakage (EL) was determined by using the protocols of (Blum and Ebercon, 1981) with slight changes. Fresh leaves (0.1 g) were detached and dipped in centrifuge tubes filled with deionized water (35 ml). The tubes were then placed on a rotary shaker for 1 day, and initial conductance (C_i_) of solutions was recorded. Afterwards, the tubes were kept in an autoclave (140°C) for half an hour, and the maximum conductance (C_m_) of the solutions was noted. EL was computed as the percentage (%) of C_i_ to C_m_. For assays of superoxide anion (O_2_^.-^) content, malondialdehyde (MDA) content, and hydrogen peroxide (H_2_O_2_) content, 0.1 g of leaf tissue was ground in 4 ml cold phosphate buffer (50 µM, pH 7.8) containing polyvinylpyrrolidone (1%, w/v). After following centrifugation at 12000 g for 30 min, the supernatant was collected. The O_2_^.-^ was examined with sulfanilamide method (Elstner and Heupel, 1976), and the absorbance of reaction solution was spectrophotometrically noted at 530 nm. The H_2_O_2_ content was estimated according to the potassium iodide (KI) protocol. The absorbance of oxidation product was noticed at 390 nm (Velikova et al., 2000). MDA content was estimated by using the procedure illustrated by (Dhindsa et al., 1981). The supernatant (0.5 ml) and reaction solution (1 ml) comprising of trichloroacetic acid (20%, w/v) and thiobarbituric acid (0.5%, w/v) were mixed and shaken thoroughly. The homogenate was placed in a high temperature water bath (95°C) for 15 min and cooled instantly by using an ice water bath. After centrifugation at 8000 g for 10 min, the supernatant was taken, and the absorbance was read at 600 and 532 nm spectrophotometrically.

### 2.5 Analysis of metabolomics

The content of different metabolites was detected by using gas chromatography-time of flight mass spectrography (GC-TOFMS). The procedure described by (Li et al., 2019a) was followed for the extraction, separation, and quantification of metabolites. A total of 20 mg of the fine dry leaf powders were mixed with 100 μL of double distilled water, and subsequently the 500 μL of aqueous methanol was added. The mixture in the tube was subjected to sonication for 20 min. Later, the centrifugation was performed at 12000 g for 10 min. Then, 300 μL of the supernatant was mixed with 10 μL of 0.3 mg/mL chlorophenylalanine (an internal standard) before desiccation in a CentriVap benchtop centrifugal concentrator (Labconco, Kansas City, MO). After being fully desiccated, the samples were reconstituted in 80 μL of methoxyamine hydrochloride and incubated at 30°C for 90 min. The 80 μL of N-methyl-N-(trimethylsilyl) trifluoroacetamide containing 1% trimethylchlorosilane was added into the mixture which was then incubated at 70°C for 60 min. The treated samples were analyzed by utilizing GC-TOFMS. The initial GC temperature was held at 80°C for 0.2 min and then increased to 180°C at a rate of 10°C min^-1^. The metabolite identification was accomplished with ChromaTOF software (v. 4.50.8.0, LECO, St. Joseph, MI, USA) and commercially available compound libraries: NIST 2005 (PerkinElmer Inc., Waltham, MS, USA), Wiley 7.0 (John Wiley and Sons Ltd., Hoboken, NJ, USA).

### 2.6 Statistical analysis

Data was arranged by using Microsoft Excel 2016 software (Microsoft Corp., Redmond, WA, United States) and figures were made by using GraphPad Prism 8.3.0 (538). Significant differences were examined with two-way ANOVA in combination with Tukey’s test at *p* ≤ 0.05.

## 3 Results

### 3.1 Effects of foliar spray of DA-6 on water status & photosynthetic functions

**Figure 1:**
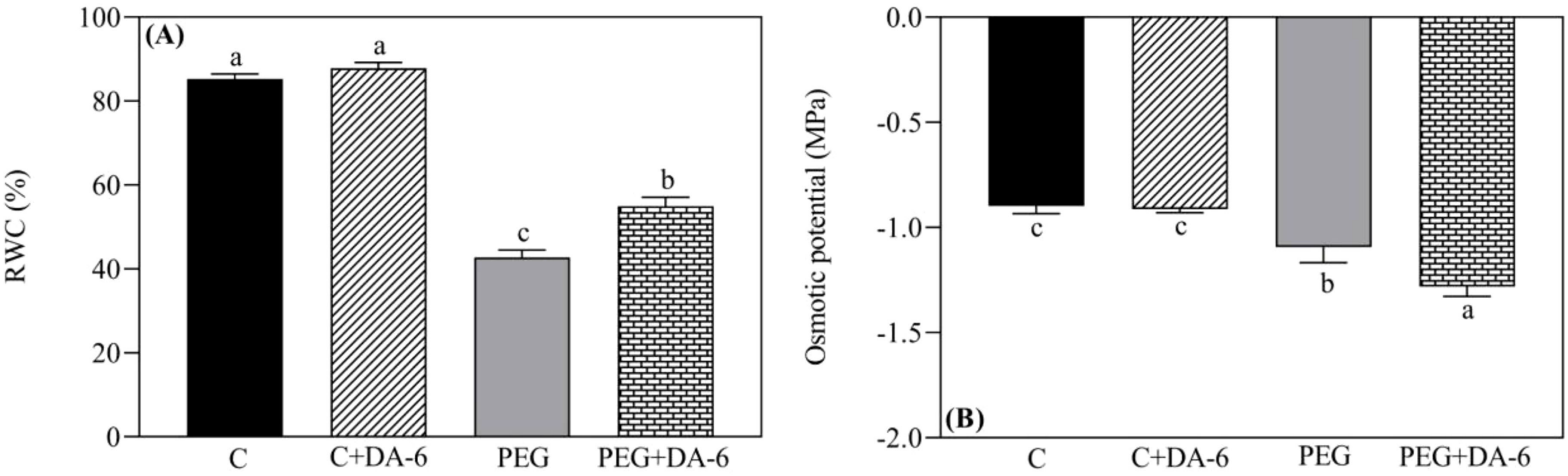
Impacts of foliar application of DA-6 on **(A)** relative water content (RWC), and **(B)** osmotic potential (OP) in leaves of white clover under normal and drought conditions. Different letters above or below the vertical columns demonstrate significant differences among different treatments based on the two-way ANOVA in combination with Tukey’s test at *p* ≤ 0.05. Vertical bars represent the ± standard error (SE) of mean (n = 4). C, well-watered control; C+DA-6, well-watered plants supplemented with DA-6; PEG, 17% PEG-induced drought stress (-0.3 Mpa); PEG+DA-6, 17% PEG-induced drought stress plus exogenous application of DA-6.

**Figure 2:**
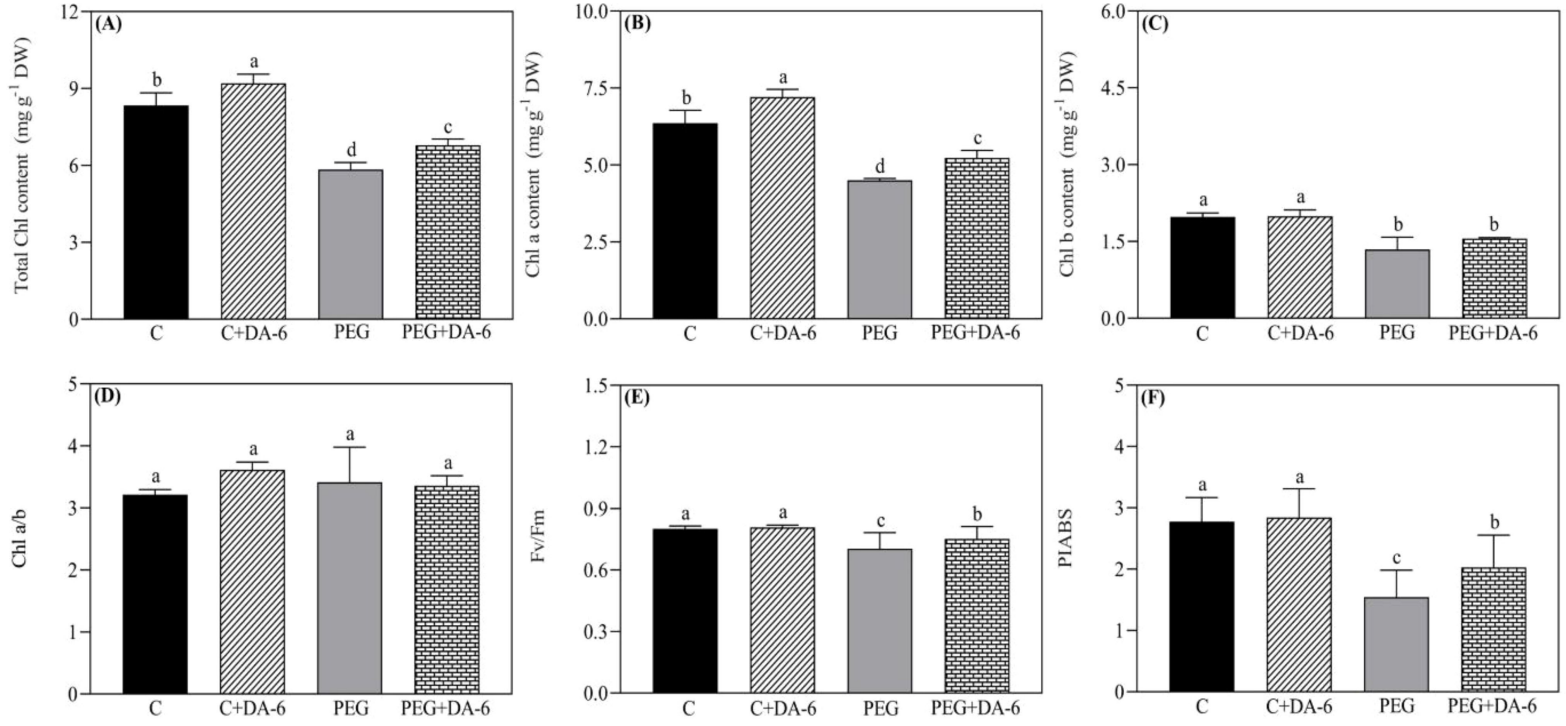
Impacts of foliar application of DA-6 on **(A)** total chlorophyll (Chl) content, **(B)** Chl a content, **(C)** Chl b content, **(D)** Chl a/b ratio, **(E)** photochemical efficiency (Fv/Fm), and **(F)** performance index on an absorption basis (PIABS) in leaves of white clover under normal and drought conditions. Different letters above the vertical columns demonstrate significant differences among different treatments based on the two-way ANOVA in combination with Tukey’s test at *p* ≤ 0.05. Vertical bars represent the ± standard error (SE) of mean (n = 4). C+DA-6, well-watered plants supplemented with DA-6; PEG, 17% PEG-induced drought stress (-0.3 Mpa); PEG+DA-6, 17% PEG-induced drought stress plus exogenous application of DA-6.

Under normal condition, foliar application of DA-6 exhibited no significant changes in RWC and OP in leaves, but white clover plants pretreated with DA-6 showed 28.48% higher RWC and 17.43% lower OP when compared with untreated plants under drought stress (**Figures 1A, B**). Exogenous DA-6 pretreatment significantly increased contents of total Chl and Chl a under non-stress condition, however, Chl b, Chl a/b, Fv/Fm, and PIABS remained unaffected by the DA-6 under normal condition (**Figures 2C–F**). Drought stress substantially reduced the total Chl content, Chl a content, Chl b content, Fv/Fm, and PIABS in both DA-6-treated and untreated plants, but did not affect the ratio of Chl a to Chl b (**Figures 2A–F**). DA-6-pretreated plants demonstrated a 16%, 16%, 7%, or 32% significantly higher total Chl content, Chl a content, Fv/Fm, or PIABS than non-treated plants exposed to drought stress (**Figures 2A, B, E, F**). The DA-6 had no significant effect on Chl b content and Chl a/b ratio under water deficient condition as shown in **Figures 2C, D**.

### 3.2 Effects of foliar spray of DA-6 on cell membrane stability & oxidative damage

**Figure 3:**
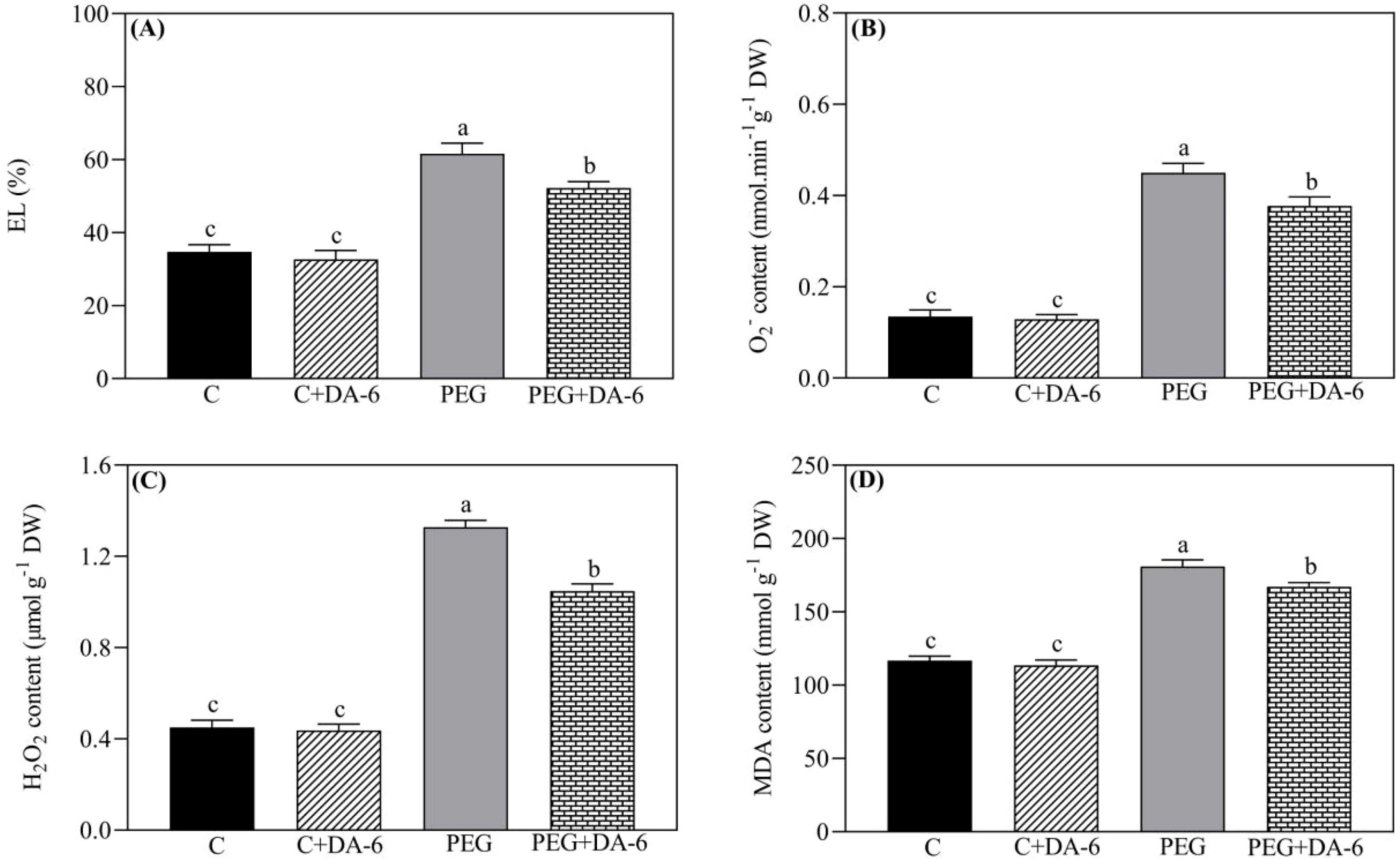
Impacts of foliar application of DA-6 on **(A)** electrolyte leakage (EL), **(B)** superoxide anion (O_2_^.-^) content, **(C)** hydrogen peroxide (H_2_O_2_) content, and **(D)** malondialdehyde (MDA) content in leaves of white clover under normal and drought conditions. Different letters above the vertical columns demonstrate significant differences among different treatments based on the two-way ANOVA in combination with Tukey’s test at *p* ≤ 0.05. Vertical bars represent the ± standard error (SE) of mean (n = 4). C+DA-6, well-watered plants supplemented with DA-6; PEG, 17% PEG-induced drought stress (-0.3 Mpa); PEG+DA-6, 17% PEG-induced drought stress plus exogenous application of DA-6.

Leaf EL, ROS (O_2_^.-^ and H_2_O_2_) contents, and MDA content were not significantly influenced by the DA-6 pretreatment under non-stressed condition as shown in **Figures 3A–D**. Drought stress significantly increased the above-mentioned four parameters in DA-6-treated and untreated white clover plants. However, foliar DA-6 application markedly reduced the drought-stimulated an increase in EL, O_2_^.-^ content, H_2_O_2_ content, or MDA content by 15%, 15.55%, 21%, or 7.60% when compared with untreated plants, respectively (**Figures 3A–D**).

### 3.3 Metabolites profiling in white clover plants influenced by foliar application of DA-6

**Figure 4:**
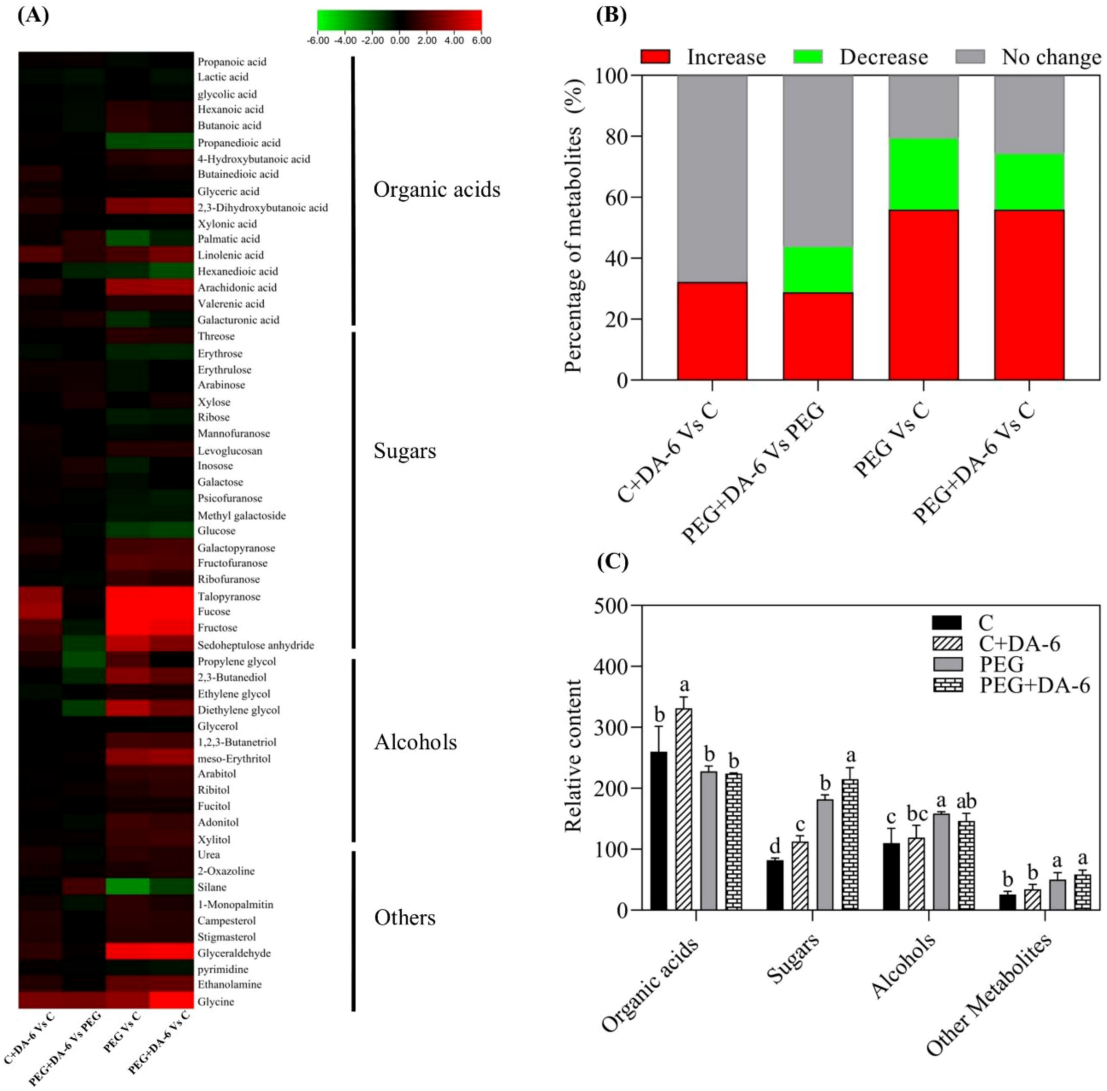
Impacts of foliar application of DA-6 on changes in **(A)** heat map of 59 identified metabolites, **(B)** percentage of unchanged, down-regulated, and up-regulated metabolites, and **(C)** relative contents of organic acids, sugars, alcohols, and other metabolites in leaves of white clover under normal and drought conditions. Different letters above the vertical columns demonstrate significant differences among different treatments based on the two-way ANOVA in combination with Tukey’s test at *p* ≤ 0.05. Vertical bars represent the ± standard error (SE) of mean (n = 4). C, well-watered control; C+DA-6, well-watered plants supplemented with DA-6; PEG, 17% PEG-induced drought stress (-0.3 Mpa); PEG+DA-6, 17% PEG-induced drought stress plus exogenous application of DA-6.

A total of 59 metabolites comprising of 17 organic acids, 20 sugars, 12 alcohols, and 10 other metabolites were detected and quantified in leaves of white clover (**Figure 4A**). Heat map of metabolites demonstrated that these metabolites were differentially regulated by foliar application of DA-6 and water stress. Majority of these metabolites remained unaffected by the DA-6 pretreatment under well-watered condition as shown by C+DA-6 Vs. C (**Figure 4B**). Under drought stress, 29% or 15% metabolites were significantly up-regulated or down-regulated by the DA-6, as depicted by PEG+DA-6 vs. PEG (**Figure 4B**). Only 24% or 19% metabolites were significantly decreased in PEG vs. C or PEG+DA-6 vs. C, respectively. In addition, a 56% upsurge in metabolites was noticed in PEG vs. C or PEG+DA-6 vs. C, respectively (**Figure 4B**). In contrast to control, drought stress significantly enhanced the accumulation of sugars and alcohols, but did not significantly affect the accumulation of organic acids in both DA-6-treated and untreated plants (**Figure 4C**). Drought stress did not induce significant effects on contents of organic acids, alcohols, and other metabolites between DA-6-pretreated and untreated plants (**Figure 4C**). However, drought stress significantly increased the accumulation of sugar in DA-6-pretreated plants when compared with non-treated plants (**Figure 4C**).

### 3.4 Differentially accumulated metabolites influenced by foliar application of DA-6

**Figure 5:**
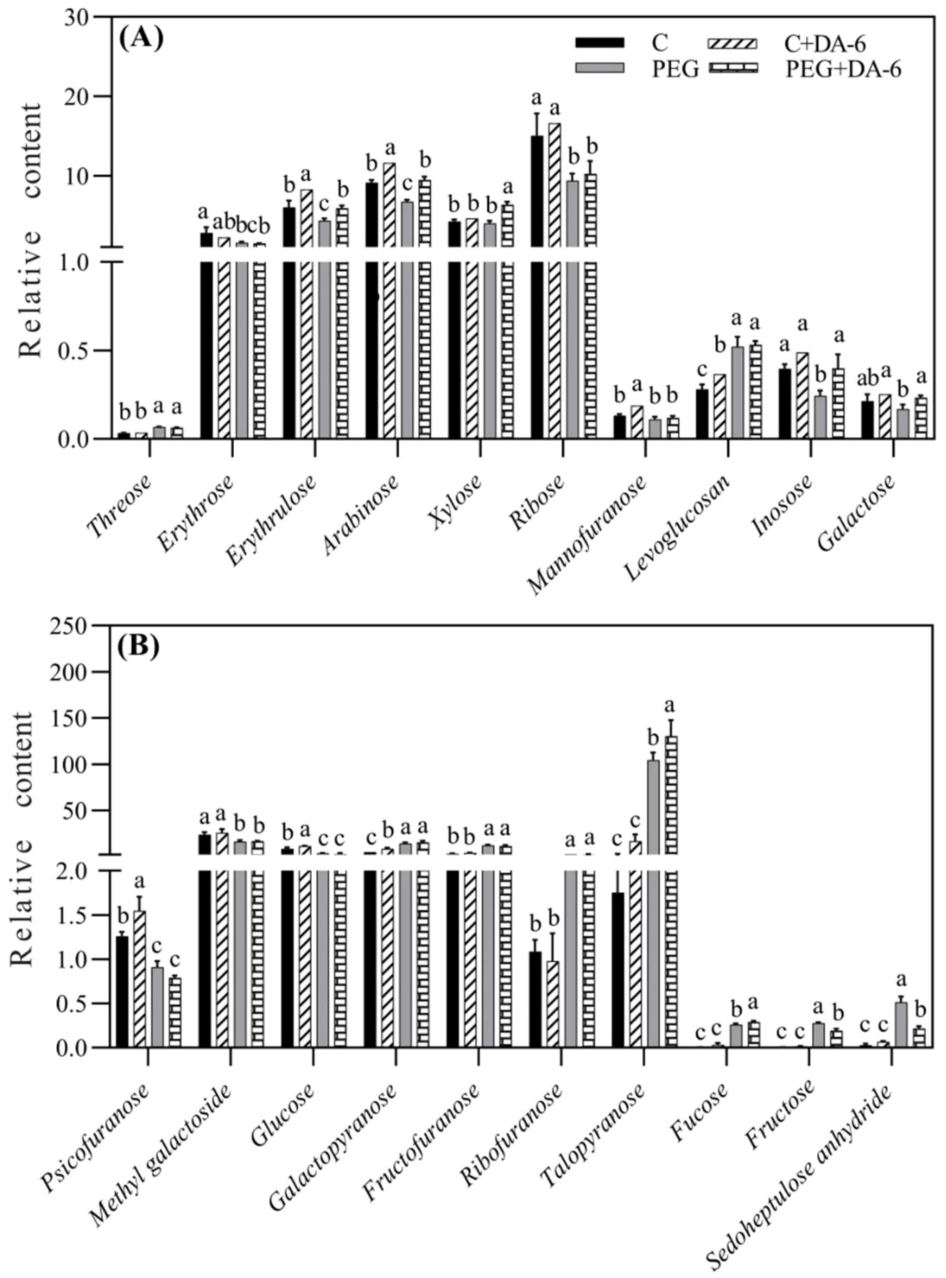
Impacts of foliar application of DA-6 on **(A, B)** sugars in leaves of white clover under normal and drought conditions. Different letters above the vertical columns demonstrate significant differences among different treatments based on the two-way ANOVA in combination with Tukey’s test at *p* ≤ 0.05. Vertical bars represent the ± standard error (SE) of mean (n = 4). C+DA-6, well-watered plants supplemented with DA-6; PEG, 17% PEG-induced drought stress (-0.3 Mpa); PEG+DA-6, 17% PEG-induced drought stress plus exogenous application of DA-6.

**Figure 6:**
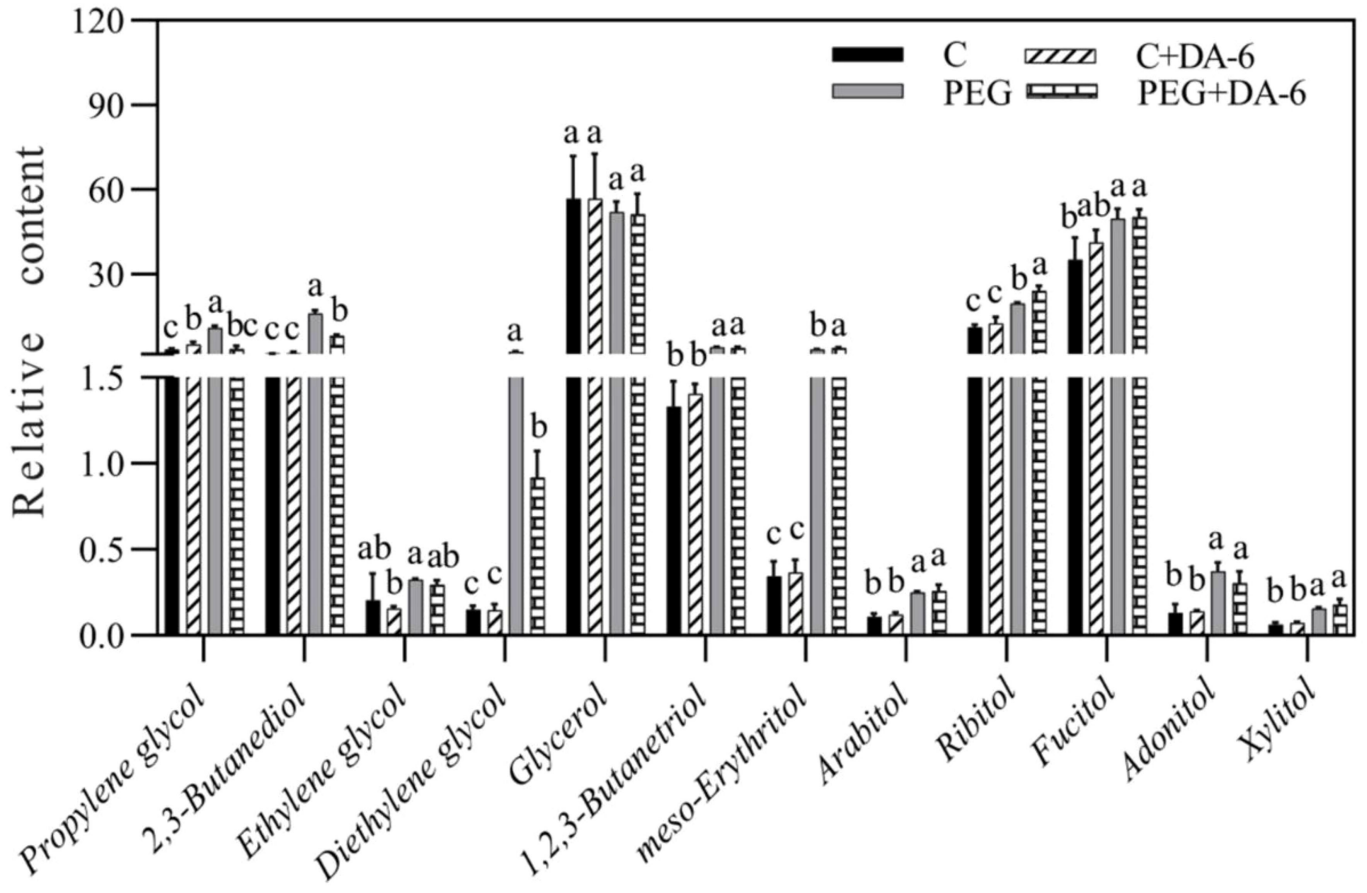
Impacts of foliar application of DA-6 on alcohols in leaves of white clover under normal and drought conditions. Different letters above the vertical columns demonstrate significant differences among different treatments based on the two-way ANOVA in combination with Tukey’s test at *p* ≤ 0.05. Vertical bars represent the ± standard error (SE) of mean (n = 4). C, well-watered control; C+DA-6, well-watered plants supplemented with DA-6; PEG, 17% PEG-induced drought stress (-0.3 Mpa); PEG+DA-6, 17% PEG-induced drought stress plus exogenous application of DA-6.

**Figure 7:**
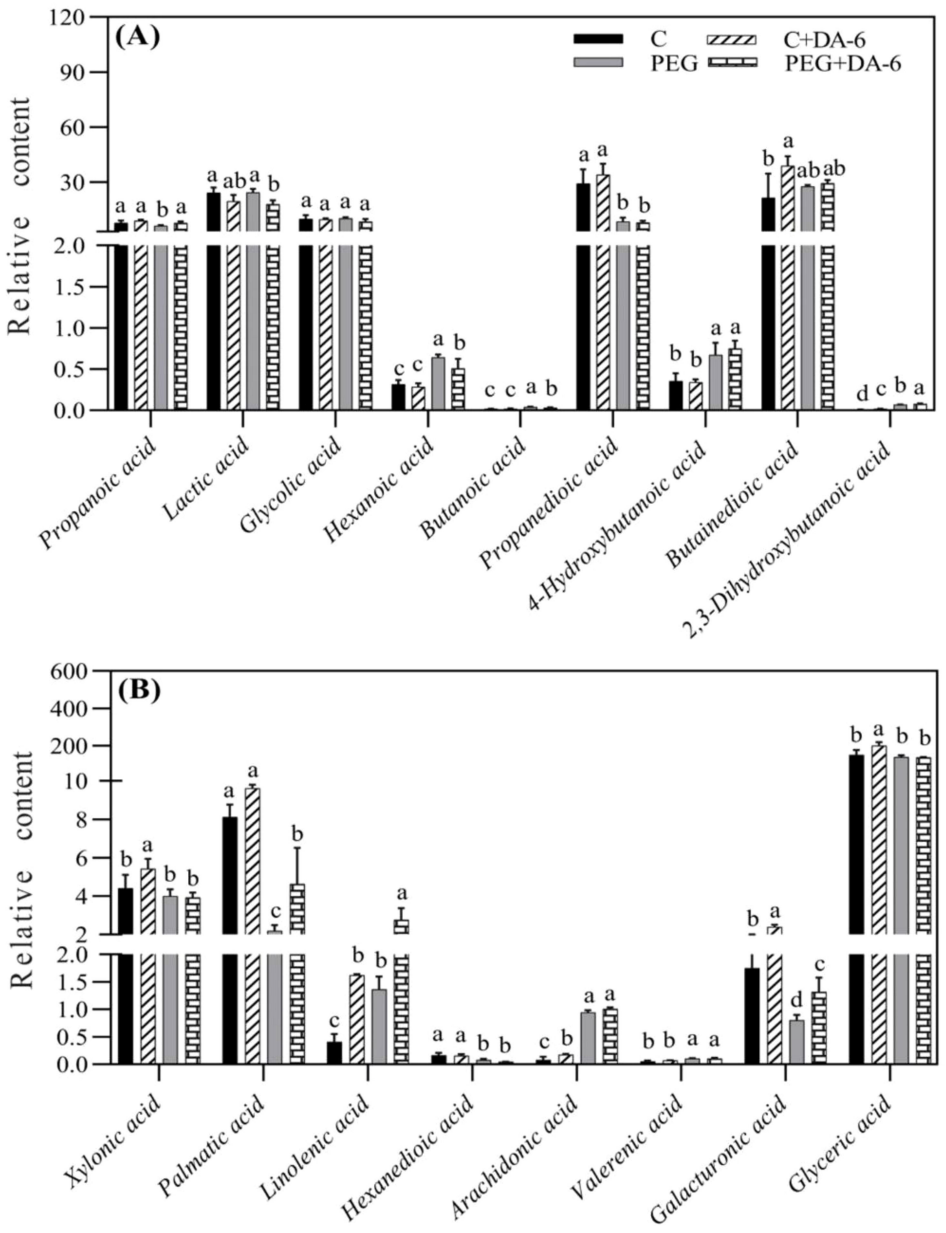
Impacts of foliar application of DA-6 on **(A, B)** organic acids in leaves of white clover under normal and drought conditions. Different letters above the vertical columns demonstrate significant differences among different treatments based on the two-way ANOVA in combination with Tukey’s test at *p* ≤ 0.05. Vertical bars represent the ± standard error (SE) of mean (n = 4). C, well-watered control; C+DA-6, well-watered plants supplemented with DA-6; PEG, 17% PEG-induced drought stress (-0.3 Mpa); PEG+DA-6, 17% PEG-induced drought stress plus exogenous application of DA-6.

**Figure 8:**
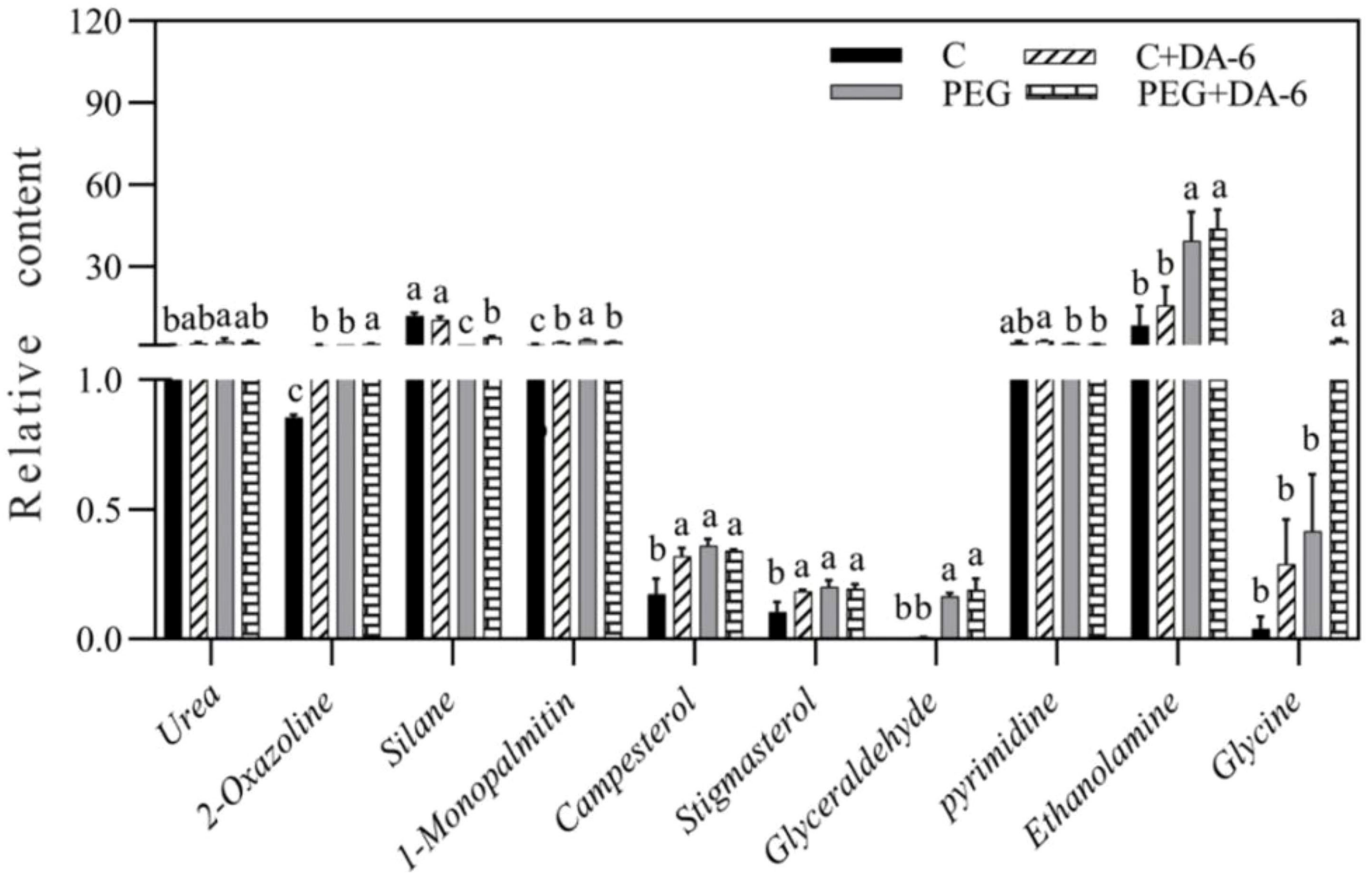
Impacts of foliar application of DA-6 on other metabolites in leaves of white clover under normal and drought conditions. Different letters above the vertical columns demonstrate significant differences among different treatments based on the two-way ANOVA in combination with Tukey’s test at *p* ≤ 0.05. Vertical bars represent the ± standard error (SE) of mean (n = 4). C, well-watered control; C+DA-6, well-watered plants supplemented with DA-6; PEG, 17% PEG-induced drought stress (-0.3 Mpa); PEG+DA-6, 17% PEG-induced drought stress plus exogenous application of DA-6.

**Figure 9:**
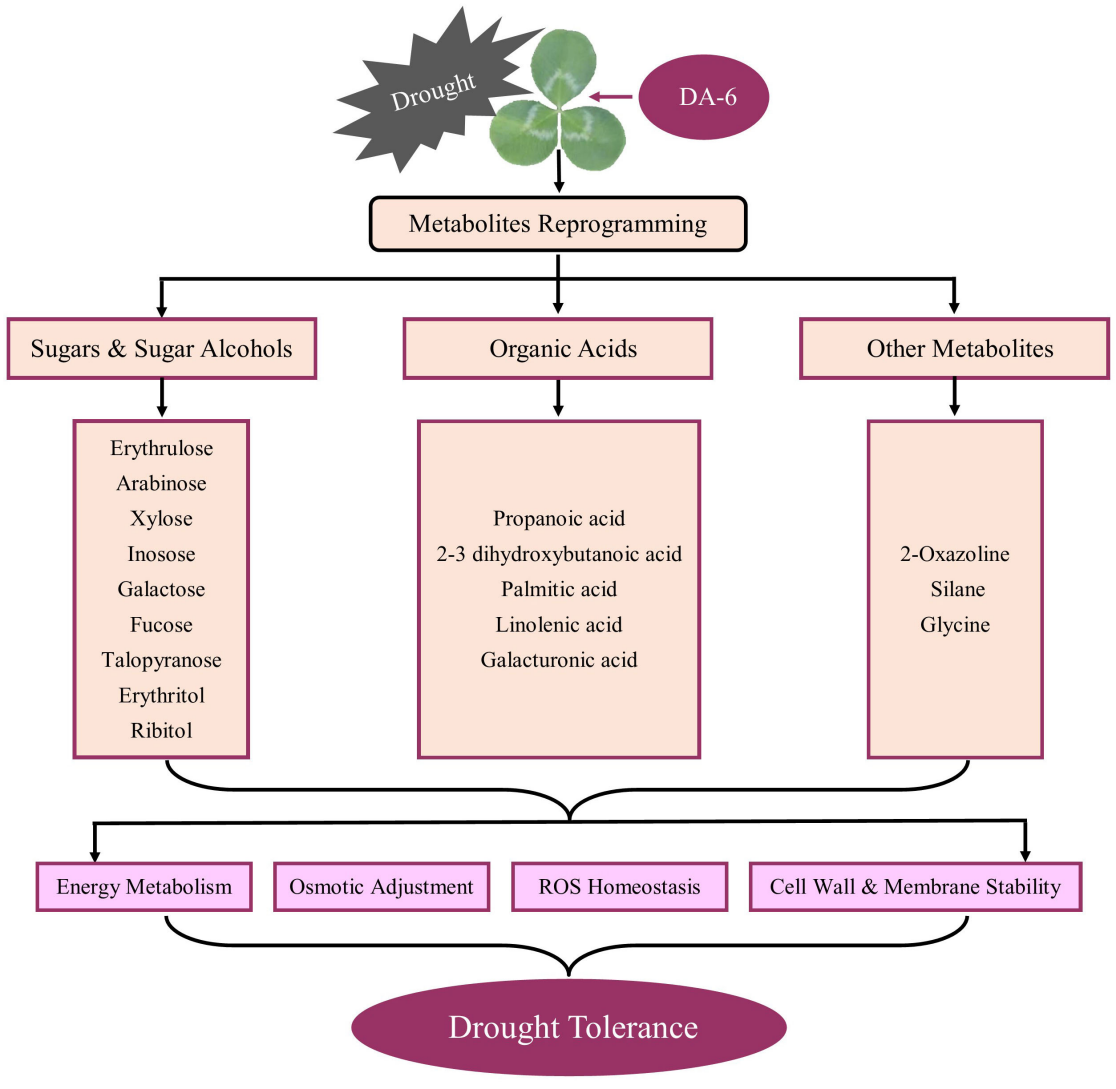
A schematic diagram illustrating DA-6-regulated metabolites reprogramming associated with adaptive response to drought stress in white clover.

Exogenous application of DA-6 significantly enhanced accumulations of multiple sugars including erythrulose, arabinose, mannofuranose, levoglucosan, psicofuranose, glucose, and galactopyranose under normal condition (**Figures 5A, B**). Under drought stress, the application of DA-6 significantly ameliorated drought-induced decreases in erythrulose, arabinose, xylose, inosose, and galactose, and also further increased contents of talopyranose and fucose (**Figures 5A, B**). In term of changes in different alcohols, drought stress significantly enhanced accumulations of 2,3-butanediol, diethylene glycol, 1,2,3-butanetriol, meso-erythritol, arabitol, ribitol, fucitol, adonitol, and xylitol in both DA-6-pretreated and non-pretreated plants (**Figure 6**). The DA-6 induced significant increases in meso-erythritol and ribitol contents under drought stress (**Figure 6**). For changes in organic acids, the PEG + DA-6 treatment exhibited significantly higher propanoic acid, 2,3-dihydroxybutanoic acid, palmitic acid, linolenic acid, and galacturonic acid contents than the PEG treatment (**Figures 7A, B**). In addition, drought stress significantly induced accumulations of campesterol and stigmasterol, and the foliar pretreatment of DA-6 significantly increased contents of 2-oxazoline, silane, and glycine under drought stress (**Figure 8**). **Figure 9** demonstrated that metabolites reprogramming related to energy metabolism, osmotic adjustment, ROS homeostasis, and cell wall and membrane stability was mediated by foliar application of DA-6 in white clover under drought stress.

## 4 Discussion

Growth retardation, leaf wilting, and senescence are the most obvious phenotypic symptoms when plants undergo water deficiency (Farooq et al., 2009). However, plants have naturally developed different adaptive strategies including hormonal regulation, OA, and antioxidant defense system to acclimatize drought stress (Seki et al., 2007; Jiao et al., 2012). Beneficial effects of the DA-6 have been reported widely in plants associated with delayed seed aging, improved photosynthesis, and enhanced antioxidant defense system and OA under normal or stressful conditions (Zhou et al., 2019; Hassan et al., 2021a). Our current study found that the foliar pretreatment of appropriate dose of DA-6 significantly ameliorated water status and photosynthetic functions of white clover leaves, as demonstrated by improved Chl content, Fv/Fm, PIABS, RWC, and OA under PEG-induced drought stress (17% PEG and -0.3 Mpa) (**Figures 1**, **2**). Moreover, DA-6-treated white clover plants also demonstrated reduced oxidative damage which was characterized by declines in contents of O_2_^.-^, H_2_O_2_, and MDA and improved membrane stability than non-treated plants exposed to drought stress (**Figure 3**). Early study of (Fu et al., 2011) found that the DA-6 application could increase tolerance of strawberry (*Fragaria ananassa*) seedlings to chilling-induced oxidative injury by enhancing antioxidant capacity and photosynthesis. Recent study of (Huang et al., 2023) also demonstrated that the DA-6 treatment effectively alleviated drought-induced oxidative damage through enhancing enzymatic antioxidant defense system in *Ananas comosus* plants. Current findings suggested a promising regulatory role of the DA-6 in drought tolerance of white clover contributing to ameliorated water balance, photosynthetic function, and cellular membrane stability. The DA-6 could be used as a potential PGR for regulating crop senescence and stress tolerance due to low cost and nontoxic character.

Stress-triggered metabolites reprograming played key roles in mediating tolerance to various abiotic stresses in plants (Patel et al., 2022). As important primary metabolites, sugars and sugar alcohols with fundamental functions of energy supply, OA, and signaling transduction are responsible for plant growth and stress defense (Bhattacharya and Kundu, 2020). Previous studies related to metabolic profiling proved advantageous effects of massive accumulations of sugars and sugar alcohols on mitigating abiotic stresses in different plant species (Shi et al., 2015; Martínez-Noël and Tognetti, 2018). Our results showed that foliar application of the DA-6 significantly induced accumulations of sugars (erythrulose, xylose, galactose, arabinose, fucose, inosose, and talopyranose) and sugar alcohols (erythritol and ribitol) in white clover after being subjected to PEG-induced drought stress (**Figures 5**, **6**). Erythrulose, xylose, and galactose are reductive monosaccharides and perform critical roles in mediating cellular metabolic homeostasis under drought stress (Li et al., 2017; Lu et al., 2023). Combined heat and drought stress resulted in higher galactose content contributing to better OA in poplar (*Populus*) leaves (Jia et al., 2016). In plants, arabinose is an essential constituent of various cell wall polysaccharides, glycoproteins, flavonoids, and signaling peptides (Rautengarten et al., 2017). Fucose has been found to be an integral component in the biosynthesis of cell wall polymers as well as sugar-regulated proteins (Reiter et al., 1997; Zentella et al., 2017). Significant increases in arabinose biosynthesis and fucose accumulation have been reported to be associated with salt tolerance in *Arabidopsis thaliana* and white clover (Zhao et al., 2019; Cheng et al., 2022). In addition, myo-inositol acting as a derivative of inosose positively regulated drought tolerance due to its roles in OA and antioxidant (Li et al., 2019b). Exogenous pretreatment of myo-inositol effectively decreased drought damage to creeping bentgrass via improvement in OA and elimination of oxidative damage (Li et al., 2020). Moreover, the positive role of erythritol and ribitol as essential osmolytes has been well documented in plants under adverse environmental conditions (Fàbregas and Fernie, 2019; Zhou et al., 2023). Our findings suggested that the DA-6-regulated drought tolerance of white clover could be associated with higher accumulation of these sugars and sugar alcohols leading to better cell wall and plasma membrane stability, OA and osmoprotection, signaling transduction, and antioxidant defense for redox homeostasis.

Various organic acids were generally or differentially regulated by DA-6 in white clover. Main functions of organic acids including pH regulation, elimination of ionic toxicity, OA, and energy metabolism have been elucidated in different plant species under unfavorable environmental conditions (Lecoeur et al., 1992; Jones, 1998; Ma et al., 2001). As crucial fatty acids, palmitic acid and linolenic acid are involved in various cellular functions such as constituents of cellular membranes, carbon and energy source in triacylglycerol, precursors of bioactive molecules, and stress signaling in plants (Zhukov, 2015; He and Ding, 2020; Hassan et al., 2022). Galacturonic acid that is an essential component of pectins in cell wall performs imperative roles in plant growth and defense system (Pourcelot et al., 2011; Kuivanen et al., 2015). Significant increases in contents of palmitic acid, linolenic acid, or galacturonic acid by the DA-6 and *β*-sitosterol pretreatment were also linked with ameliorated drought tolerance of white clover (Li et al., 2019a; Hassan et al., 2022), which is in accordance with our present findings. The DA-6-mediated increases in palmitic acid, linolenic acid, and galacturonic acid could be associated with improved lipids reprograming, therefore resulting in superior cell membrane and wall stability under water-limited condition. In addition, the DA-6 application also induced accumulations of propanoic acid and 2,3-dihydroxybutanoic acid in white clover leaves in response to PEG-induced drought stress. However, regulatory roles and mechanisms of short chain fatty acids propanoic acid and 2,3-dihydroxybutanoic acid in stress tolerance have rarely been reported in plants so far and still demand an in-depth investigation in our future studies.

Apart from sugars, alcohols and organic acids, other metabolites such as 2-oxazoline, silane, and glycine also responded to the DA-6 differentially in white clover under PEG-stimulated drought stress (**Figure 8**). The 2-oxazoline is a heterocyclic organic compound for the biosynthesis of complex oxazolines which have potential roles in protecting carboxylic acids (Wenker, 1938). Various 2-oxazolines such as 2-ethyl-2-oxazoline perform living cationic ring-opening polymerization to produce poly(2-oxazoline)s (Kobayashi and Uyama, 2002; Hoogenboom, 2009). A recent study reported the synergistic role of biochar and poly (2-ethyl-2-oxazoline) hydrogels in improving carrot (*Daucus carota*) production under saline condition (Abdeen et al., 2023). Silane serves as a precursor in the synthesis of elemental silicon. The beneficial role of silicon in morphological, physiological and biochemical functions of plants has been well documented under different abiotic stresses including drought (Luyckx et al., 2017; Zargar et al., 2019). In addition, silanes possessing inorganic or organic attachments are coupling agents and adhesion promoters (London et al., 2013). Glycine performs vital roles in amino acids metabolism, signaling as well as plant stress responses (Depeint et al., 2006). The study of (Li et al., 2019b) reported that exogenous application of γ-aminobutyric acid significantly enhanced the glycine accumulation in favor of amino acids homeostasis in white clover seedlings under drought stress. Significant upsurge in the accumulation of glycine was also associated with improved thermo-tolerance of creeping bentgrass (Li et al., 2016). These findings indicated that the DA-6-regulated adaptability to PEG-stimulated drought stress could be connected with increased accumulations of 2-oxazoline, silane, and glycine in leaves of white clover plants. However, functions of 2-oxazoline associated with drought tolerance could not be fully explained in our current study and deserves to be further studied, since little information is available so far.

Interestingly, PEG-induced drought stress significantly induced accumulation of campesterol and stigmasterol in both of DA-6-pretreated and untreated white clover plants, and the DA-6 application also increased their accumulations in leaves under normal condition. Campesterol and stigmasterol are key regulators of plasma membrane fluidity and integrity as components of the phospholipid bilayer membrane in plants (Saffan, 2008). *OsFes1A*-transgenic *A. thaliana* with a significant increase in endogenous campesterol content exhibited significantly higher drought tolerance than wild type (Xu et al., 2024). Both of drought-sensitive and drought-tolerant rice (*Oryza sativa*) cultivars could significantly accumulate compesterol and stigmasterol for better maintenances of membrane lipids homeostasis and the integrity of cell membranes in response to drought stress (Kumar et al., 2018). Higher accumulation of phytosterols such as campesterol and stigmasterol as well as better membrane stability during drought stress were observed in drought-tolerant rice genotype as compared to drought-sensitive one (Kumar et al., 2015). On the contrary, barley (*Hordeum vulgare*) genotype with the lowest level of campesterol was susceptible to drought stress (Kuczyńska et al., 2019). In addition, a previous study reported that heat-tolerant hard fescue (*Festuca trachyphylla*) genotype Reliant IV demonstrated higher stigmasterol content when compared with the heat-sensitive Predator (Wang et al., 2017). Exogenous stigmasterol mitigated negative impact of drought stress on flax (*Linum usitatissimum*) plants through activating antioxidant defense to relieve oxidative damage (Hassan et al., 2021b; Nemat Alla et al., 2022). Recent study of Hanafy and Sadak also found that foliar application of stigmasterol could significantly improve growth and productivity of sunflower (*Helianthus annuus*) related to enhanced antioxidant metabolism (Hanafy and Sadak, 2023). Based on these previous reports and our current study, the accumulations of campesterol and stigmasterol could be important adaptive responses to drought stress in white clover due to beneficial roles of campesterol and stigmasterol in regulating cell membrane stability and antioxidant defense.

## 5 Conclusion

Exogenous application of DA-6 significantly alleviated drought-induced oxidative damage and also improved water balance, photosynthetic function and cell membrane stability when white clover suffered from drought stress. Metabolic profiling demonstrated that a total of 59 metabolites were generally or differentially regulated by the DA-6 under water-limited condition. The DA-6 induced the accumulation of sugars and sugar alcohols including erythrulose, arabinose, xylose, inosose, galactose, talopyranose, fucose, erythritol and ribitol. In addition, foliar pretreatment of DA-6 also significantly enhanced the accumulation of various organic acids and amino acids such as propanoic acid, 2,3-dihydroxybutanoic acid, palmitic acid, linolenic acid, D-glucuronic acid, lactone, and glycine. The DA-6-induced increases in these organic metabolites could be contributed to improved drought tolerance of white clover due to their potential roles in OA, osmo-protection, ROS homeostasis, cell wall structure and membrane stability. The present findings provide new evidence of DA-6-regulated metabolic homeostasis related to drought tolerance in leguminous plants.

## Data Availability

The original contributions presented in the study are included in the article/supplementary material. Further inquiries can be directed to the corresponding authors.
